# Advancing Policy and Practice in Medicaid Home and Community-Based Services Quality

**DOI:** 10.3389/fresc.2022.876871

**Published:** 2022-06-02

**Authors:** Joseph Caldwell, David Machledt

**Affiliations:** ^1^The Lurie Institute for Disability Policy, Brandeis University, Waltham, MA, United States; ^2^National Health Law Program, Washington, DC, United States

**Keywords:** Medicaid, quality, United States, long-term services and supports (LTSS), home and community-based services (HCBS)

## Abstract

This policy brief highlights recent developments and future directions in the Medicaid Home and Community Based Services (HCBS) quality policy and practice within the US. Background is provided about the structure of Medicaid HCBS within the US, the changing landscape of payment and service delivery, and implications for HCBS quality measurement and use. An overview of a HCBS quality framework is provided that was developed with stakeholder input. Frequently used survey tools, existing quality measures, and measure development are discussed. Actionable recommendations are made, including establishment of stakeholder input mechanisms, enhanced federal guidance on a core set of measures, improved data collection and stratification to address equity, multiple mechanisms to assess quality, and increased federal investment in HCBS quality infrastructure.

## Background

Long-term services and supports (LTSS) refers to a wide range of health and social services provided to individuals who need help with personal care tasks or activities of daily living, such as eating, bathing, and dressing, or with instrumental tasks, such as medication management, meal preparation, and supports for community participation and employment (1). The US lacks a coordinated and comprehensive finance and delivery system for Long Term Supports and Services (LTSS). Though an estimated 14 million US residents need LTSS, the vast majority do not receive paid services and supports (2, 3). Most rely on unpaid supports from family and friends. Among individuals with LTSS needs residing in the community, only approximately 13% receive any form of paid assistance (3).

Medicaid is the primary funder of formal LTSS in the US (4, 5). It is a joint federal (national) and state medical assistance program for low income individuals. Medicaid has strict income and asset eligibility limits. Many people with disabilities have to limit their income or spend down assets just to obtain needed services and supports. Moreover, because the program is a federal-state partnership, eligibility and benefits covered vary considerably across states.

Historically, Medicaid's structure generated an institutional bias for LTSS. Institutional care was the first type of LTSS that Medicaid covered and remains mandatory for states to provide. Over the decades, Medicaid has been a major source of funding for innovations that provide services in homes and other community-based settings. People with disabilities and older adults almost universally prefer Home and Community-Based Services (HCBS) to institutional LTSS, and they are also typically cheaper to provide and lead to better outcomes. But they remain optional Medicaid services. Moreover, the principal authority states use to design their HCBS programs – the 1915(c) HCBS waiver – permits states to impose enrollment and budget caps that other optional Medicaid services do not have. Section 1915(c) allows states to “waive” certain general Medicaid requirements to target HCBS toward specific populations, so long as beneficiaries have an institutional level of care need. Also, unlike other Medicaid eligibility categories, 1915(c) also allows states to cap enrollment and cap individual service budgets. State governments like these features because they make it easier to manage Medicaid costs, though that may come at the expense of providing access to needed services for all their residents. Some states maintain long waiting lists for these programs, meaning that some people with disabilities and older adults must wait years to access HCBS even though they could enter a nursing facility immediately (6).

Over time the share of Medicaid LTSS expenditures spent on HCBS has increased. HCBS expenditures exceeded institutional expenditures in 2013 and reached 59 percent in 2019 (4). Despite significant expansion and innovation in Medicaid HCBS over the past several decades–from promoting self-directed services, where individuals can hire and train their own care workers, to supporting competitive-integrated employment–access to HCBS for people with disabilities varies widely between states and across populations. As a joint federal-state program, Medicaid allows states substantial discretion to define their LTSS programs (6). States typically operate multiple HCBS programs targeted at different populations (e.g., developmental disabilities, acquired brain injury, physical disabilities and older adults) through a patchwork of different Medicaid authorities that each have different requirements. States utilize different delivery systems and cover different services (7). Even among those already receiving HCBS, substantial unmet needs remain common (8).

## Building Quality into Medicaid HCBS Programs

Medicaid's federal-state partnership and historical institutional bias have also shaped approaches to measuring and monitoring quality within Medicaid HCBS programs. The federal government has long regulated nursing facilities and has created a fairly robust national system for reporting on health and safety, staffing hours, and quality metrics (9). But the infrastructure around Medicaid HCBS quality has received far fewer resources and, consequently, is much less developed. Though the Centers for Medicare and Medicaid Services (CMS) has issued federal regulations and guidance, states still largely determine how they approach quality within their HCBS programs. The resulting variability complicates the creation of a coordinated federal approach to HCBS quality.

Quality oversight in 1915(c) waivers amounts to states attesting to six broad assurances in their waiver application, three of which relate to care quality:

1. The state ensures that all waiver services are provided by qualified providers (through licensure or certification standards, monitoring, and oversight over training methodologies).
2. The state shows it has an effective system for reviewing the adequacy of participants' service plans (including choice of providers, regular updates, comprehensiveness).
3. The state shows it has an effective system for assuring participant health and welfare with mechanisms to prevent abuse and neglect, regulate use of restrictive interventions, manage critical incidents, and establish overall health care standards (10).

Since these assurances were added to the 1915(c) waiver approvals in 2004, states have managed their own oversight systems for 1915(c) HCBS programs, with CMS stepping in occasionally to implement corrective action plans if the state has not met its assurances. Most of the state-reported measures focus on whether appropriate processes are in place. CMS reviews the state systems during waiver renewals or applications, and asks its regional offices to conduct onsite reviews at least once over the course of each 3–5-year waiver period. Prior reports and investigations from the Government Accountability Office (GAO) have found that even CMS's limited quality reviews showed that many states did not have adequate systems to meet all three quality assurances, and some received reapprovals despite failures to correct the problems CMS identified (11). More recent GAO studies found similar shortcomings in HCBS quality in capitated managed care (see below) (12).

Other HCBS covered outside 1915(c) programs receive even less scrutiny. While acute care and preventive services like emergency room care, immunizations, and diabetes control are well represented in Medicaid's core measure sets for children and adults, HCBS long lacked any nationally-reported measures. Until 2014, there was not even a meaningful federal Medicaid definition of what can qualify as home or community-based setting.

## Quality and Accountability in Managed Long-Term Services and Supports

The landscape of HCBS has rapidly changed over the past two decades. Many states have shifted away from fee-for-service (FFS) payment models to new LTSS delivery systems. The FFS system pays providers for each service provided, and has been criticized for rewarding duplicative or unnecessary services. Managed care claims to deliver care more efficiently by improving coordination and information management and restricting provider networks. The most common managed care delivery system replaces FFS with a risk-based, capitated model, where managed care organizations (MCOs) receive a fixed per member/per month payment. This incentive structure financially rewards managed care plans that spend *less* on care (at least in the short term). If a plan's health care expenditures are lower than the fixed monthly payment, the MCO keeps the remainder as profit. Importantly, without effective mechanisms to monitor and evaluate care quality and access, capitated managed care replaces the perceived fiscal excess of FFS with a system that could encourage plans to denying or delaying medically necessary care to save money.

The managed Long-Term Services and Supports (MLTSS) model has grown rapidly in the US (13). In 2004, only eight states had any MLTSS program, and enrollment of MLTSS users barely exceeded 100,000 individuals nationwide (14). By July 2019, 24 states had implemented capitated MLTSS programs, with several pending (15). Total enrollment has surpassed 1.8 million individuals (16). The most common populations served in MLTSS programs have been older adults and adults with physical disabilities. However, more recently some states have incorporated individuals with intellectual and developmental disabilities (I/DD) in statewide MLTSS or developed MLTSS programs specifically for this population (13, 17).

The growth of MLTSS has fueled renewed interest in HCBS quality measurement. With proper design and oversight, states and advocates can use quality and performance measures as one tool to achieve desired outcomes. Contracts increasingly include payment incentives tied to quality outcomes. For example, some states withhold a portion of the capitated rate contingent upon an MCO meeting certain performance metrics. Many states have incentivized shifting expenditures from institutional LTSS to HCBS through MLTSS programs (18, 19). Others have attempted to incentivize community employment outcomes through MLTSS (17). But effectiveness of these “pay for performance” incentives is contingent on valid and reliable HCBS quality measures. Moreover, these approaches alone are insufficient to ensure consistent access to high quality care.

The 2016 CMS Managed Care Rule–the first major update of Medicaid managed care regulations since 2002–issued new requirements for states and MCOs in the area of quality. This included new requirements to validate provider network adequacy annually, to describe the state's plan to reduce health inequities, and to create a new Quality Rating System for Medicaid managed care plans. The update also incorporated new protections specific to MLTSS. As of July 2017, states with MLTSS programs are required at a minimum to report measures related to quality of life, shifting expenditures from institutional to HCBS, community integration activities, and whether beneficiaries receive the services and supports set forth in their care plans.

Unfortunately, many of the 2016 regulations have taken years to implement. The proposed Quality Rating System has not yet been released for public review and comment. States still await CMS guidance on how to implement network adequacy validations, and so have not been required to do it. Annual reports of each managed care plan, including MLTSS plans, that will detail grievances, financial performance, and other metrics will only finally be required beginning after July 2022 (20). So while the regulations have taken steps to advance quality reporting and accountability for Medicaid HCBS, many gaps remain (21).

## HCBS Quality Framework

As more states shifted to MLTSS, advocates expressed concerns about the MCOs' frequent poor understanding of the person-centered, non-medical nature of HCBS (22). Moreover, the field of HCBS quality measurement lagged far behind measure development and implementation for acute care and medical settings. By 2015, the National Quality Forum (NQF) – an independent organization that brings stakeholders together to review and endorse performance measures used by the government, states, and private-sector organizations–had endorsed at most a handful of quality measures specific to HCBS.

**Figure 1:**
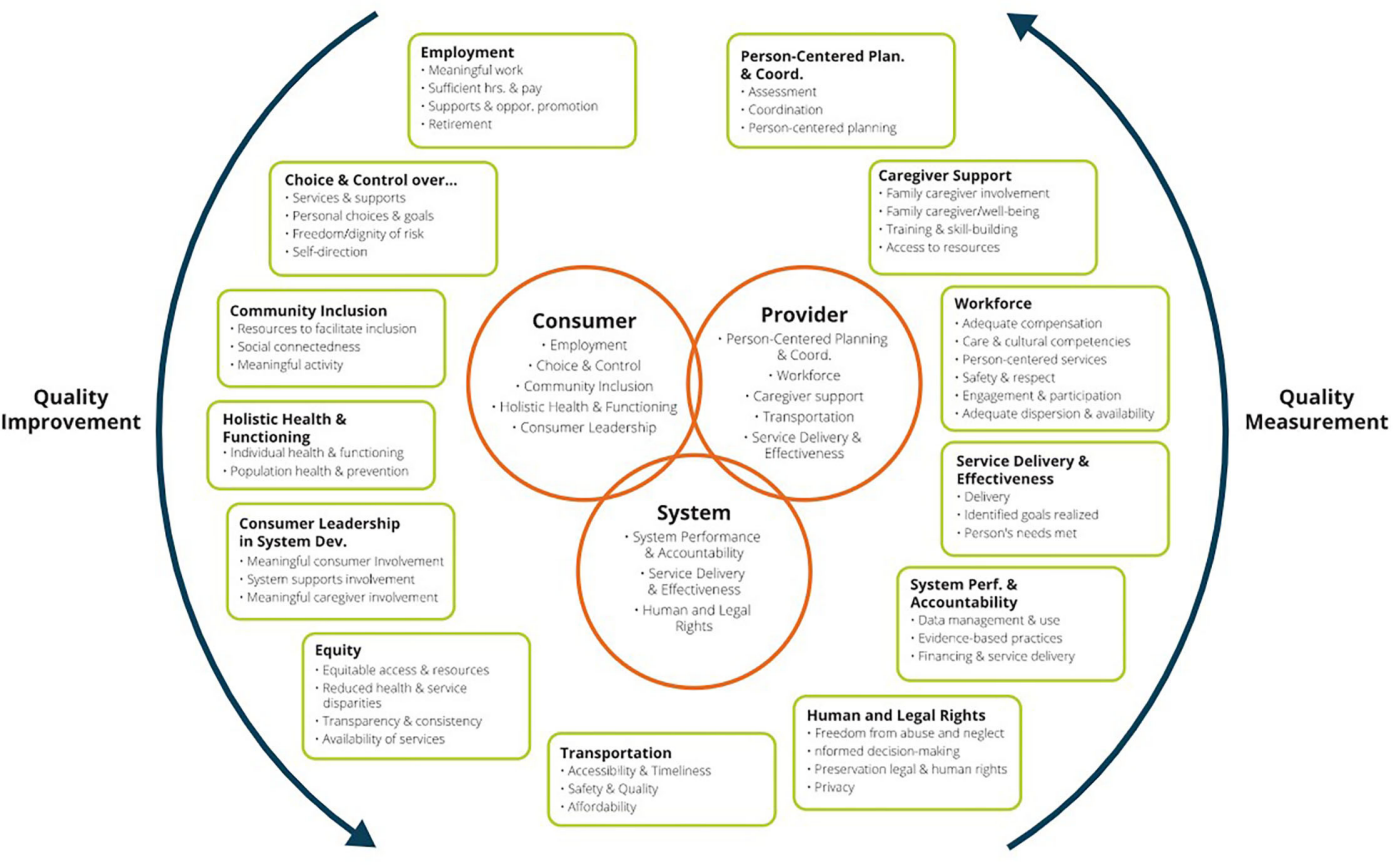
National Quality Forum Framework on HCBS Quality (25).

In response to these concerns, the Administration for Community Living (ACL) and CMS sponsored NQF to convene a multi-stakeholder workgroup to develop a HCBS quality framework, identify gaps, and make recommendations for new measure development. Twenty-two participants, including individuals with disabilities, aging and disability advocates, researchers, and representatives from providers, states, and health plans routinely met for over a year and developed an operational definition of HCBS and a quality framework consisting of 11 domains and forty subdomains (23). The University of Minnesota then conducted follow-up focus groups with 320 participants to assess the framework, including perspectives from across the disability community (24, 25). They generally validated the NQF framework and recommended inclusion of some additional subdomains (See Figure 1).

In addition to the HCBS quality framework, the NQF Committee identified potential measure concepts within each domain and made recommendations for measure development. This spurred additional federal investments through ACL and CMS. While the HCBS quality framework is specific to the US system, many domains are in alignment with the core components of the World Health Organization Building Blocks for health systems, particularly the areas of service delivery and workforce (26).

## State of HCBS Survey Instruments and Measures

The Rehabilitation Research and Training Center on HCBS Outcome Measures (RTCOM) at the University of Minnesota developed a database of over 130 instruments that have been used to measure HCBS outcomes (https://rtcom.umn.edu/database). The database was organized around the NQF domains. Most of the instruments are survey tools that cover a wide range of domains. Four of the most frequently used instruments that measure experience and person-reported outcomes are detailed in Table 1. Each has advantages and disadvantages: they differ in target populations, rules for use of proxy responses, survey administration method, and response rates.

**Table 1:** Most frequently used HCBS instruments.

**National core indicators (NCI)**
NCI is a collaboration between the Human Services Research Institute (HSRI), National Association of State Developmental Disabilities Directors (NASDDDS), and participating states (27). Started in 1997, 46 states and the District of Columbia have participated. The in-person survey of adults with developmental disabilities receiving Medicaid HCBS includes approximately 100 indicators across five domains. In 2019, CMS incorporated NCI into its overall Adult Core Set. In 2021, NQF endorsed a subset of measures from NCI.
**National core indicators -aging and disabilities (NCI-AD)**
In 2012, HSRI, ADvancing States, and participating states began adapt NCI to help evaluate quality for people with physical disabilities and older adults receiving HCBS (28). The resulting instrument, NCI-AD, began its first round of data collection in 2015-16. Twenty-nine states have participated to date. The in-person survey consists of approximately 50 core indicators across 18 domains.
**HCBS consumer assessment of healthcare providers and systems (CAHPS) survey**
CMS developed the HCBS CAHPS survey for use by states in fee-for-service and MLTSS (29). It consists of 69 core items that ask beneficiaries to report on their service experience, including getting needed services, communication with providers, case management, community inclusion and empowerment and choice of services. There is also a supplemental employment module. The survey is conducted in-person or over the telephone. In November 2016, NQF endorsed 19 measures from the instrument. To date, only a handful of states have used the HCBS CAHPS, but CMS is promoting greater adoption through a learning collaborative and technical assistance.
**Council on quality and leadership (CQL) personal outcome measures (POMs)**
The Council for Quality and Leadership (CQL) developed the POM tool in 1992 (30, 31). The POMs is an individual-level discovery tool used to determine what is important to the person receiving supports. It includes 21 indicators across 5 factors (My Human Security, My Relationships, My Community, My Choices, My Goals). CQL–trained interviewers conduct each in person interview. The POMs tool has primarily been used at the provider level, mostly with providers providing services to individuals with intellectual and developmental disabilities, as part of an accreditation process.

Despite these differences, some factors affecting quality measurement apply across instruments. For example, in-person interviews require more resources than measures using administrative or claims data. This can deeply influence the extent to which states use these instruments to improve quality. For example, NCI and NCI-AD (the most frequently used instruments) have typically been implemented with small sample sizes, around 400 individuals. This can flag general areas of strength or concern at a state or systems level, but may not provide the state with detailed data to inform corrective actions or to identify inadequate service quality for individuals or subpopulations of interest.

Some states MLTSS programs have used these survey tools and HCBS CAHPS with larger samples to enable valid analysis at the MCO level. Minnesota, for example, conducted over 2100 surveys of roughly 20,000 HCBS recipients with developmental disabilities for its last in-person data collection in 2019 (37). The CQL POMs instrument, on the other hand, has mostly focused on the provider level. This can provide very actionable data, but may not generate a representative sample to learn about trends or problems at the plan or state level. Either way, the cost and time involved in conducting surveys has definitely limited some of the impact of these early HCBS measures.

The federal government has funded additional measure development to fill gaps identified by the NQF Committee. CMS contracted to develop eight measures that include LTSS assessment, care planning, falls prevention, and rebalancing (reducing admissions to institutions, minimizing length of stay, and transitions from institutions to the community) (10). Several of these measures have now been formally endorsed by NQF and included in CMS's proposed HCBS Recommended Core Set. Some are now being used by the National Committee for Quality Assurance (NCQA) in their accreditation of MCOs providing MLTSS (32). CMS is currently contracting to re-specify some of these measures for broader application and develop additional measures in other domains where there are gaps, such as workforce and caregiver support. The RTCOM, funded by the National Institute on Disability, Independent Living, and Rehabilitation Research (NIDILRR) within ACL, is also developing new person-reported measures in areas including employment, meaningful activity, transportation, social connectedness, and choice and control.

## Policy and Practice Recommendations

While substantial progress has occurred over the past decade in HCBS quality measurement, there is still a long way to go. While maintaining the federal-state partnership that promotes innovation, we call for a stronger federal role in reporting, oversight, transparency and investment in meaningful use of measures to enhance quality and address equity.

### Establish Regular Stakeholder Input Mechanisms for HCBS Quality at the Federal and State Levels

At the federal level, the Secretary of Health and Human Services should establish a multi-stakeholder HCBS Quality Committee that centers representation on the diverse array of people receiving, or in need of Medicaid HCBS and representatives of aging and disability advocacy organizations. Other stakeholders should include the major players in quality measurement, such as health plans, measure developers, measure steward organizations, provider representatives, and states and relevant national associations representing state officials. The quality committee could help define and regularly update a core HCBS quality measure set (discussed below), inform measure development to fill gaps in measures, and act as an advisory body for other elements of HCBS quality at the national level.

States should also be required to establish their own HCBS Quality Committees based on a similar structure as the federal committee. A handful of states have already established such entities indicating feasibility. States would have flexibility to build upon existing committees and coordinate with other requirements, such as those in the managed care regulations. For example, every state has a Medicaid Medical Care Advisory Committee (MCAC) that includes beneficiaries, advocates, providers, and state officials that could be a basis for an HCBS Quality committee. Alternatively, in MLTSS programs, each plan is required to establish and maintain a member advisory committee with a representative sample of the LTSS population that could be a source for a state quality committee. Of course, the existence of quality committees should not supplant opportunities for public comment on selecting reportable measures, developing quality strategy priorities, and so forth. However, this structure would allow states and health plans to benefit from sharing the lived experience of people who use the HCBS system, while improving transparency and allowing beneficiaries to build up expertise in a technical field that plays a vital oversight role in HCBS quality.

### Establish a Core Set of HCBS Quality Measures and Require Transparent Public Reporting

The federal government should issue guidance on a core and supplemental set of HCBS quality measures. CMS began work on this in 2020 through issuing a public request for information (33); additional work is needed to finalize and incentivize rapid state implementation of both core and supplemental sets. Ultimately, states should be required to publicly post annual reports on all the core measures. Recent legislation has already mandated reporting on Medicaid and CHIP core sets for children (34) and for behavioral health measures (35) starting in 2024. Required core measures should set a federal minimum for quality oversight of HCBS to facilitate the creation of national benchmarks and apples-to-apples comparisons across states. However, CMS should continue to support state innovation to develop and use additional HCBS measures that fill gaps or allow for easier administrative reporting.

States should also publicly report HCBS quality data in ways that allow beneficiaries to compare quality across HCBS programs, managed care plans, and even providers. Public reporting on HCBS quality at the plan (and eventually provider) level could help individuals and families to make informed choices to suit their care needs.

### Improve Data Collection and Require Stratification to Address Equity in HCBS Quality

The COVID-19 pandemic has reemphasized the longstanding structural inequities in the US healthcare system. Moreover, the pandemic has exposed major holes in our data systems that make it hard to even identify health disparities, let alone inform effective remediation. The pandemic has reenergized a push to build data systems that can collect, report, and verify data stratified by key demographic factors including by race, ethnicity, disability status, age, sex, sexual orientation, gender identity, race, ethnicity, primary language, rural/urban environment, and service setting. The systems must permit analysis across multiple demographic categories, such as race and disability, so we can track compound disparities and then focus resources on improving them. Stratification should not only apply to HCBS core measures, but also allow us to know more about disparities people with disabilities may experience accessing preventive and acute care services, such as diabetes-control or vaccinations.

### Enhance Oversight and Accountability Through a Multi-Faceted Quality System

An effective quality control system for HCBS systems must include multiple pathways to evaluate services. Though performance measures provide important insights about HCBS quality and access, they cannot provide a complete picture across the range of services and providers covered. Survey samples may not be big enough, or the time lag from data collection to reporting may be too great to catch incipient problems. For this reason, HCBS quality systems must include other accountability mechanisms that use different methods, For example, each state should designate an HCBS Ombuds office charged with both helping beneficiaries troubleshoot problems using the HCBS program and with rapidly identifying and publicly reporting common problems to direct system improvements. States and plans could also track and report data on grievances and appeals to flag potential problems that may not be reported in the array of performance measures. A similar approach has already been piloted in multiple states that participated in a Medicare/Medicaid integration demonstration focused on older adults and people with disabilities (36).

### Increase Federal Investment to Improve HCBS Quality Infrastructure

The growth of MLTSS only sharpens the urgency for Congress to significantly increase the federal funding for administrative activities related to adoption of HCBS quality activities. This includes consumer and other stakeholder engagement, data and quality infrastructure, expanding the sample size for beneficiary experience surveys, and facilitating public, stratified reporting of quality measures. Additional federal funding, such as an enhanced federal match for expenditures related to HCBS quality improvement, could accelerate development of new quality measures to fill gaps, such as workforce and employment metrics, that could help to overcome the institutional bias in Medicaid quality measurement. CMS must provide ongoing technical assistance activities to states in meaningful use of measures to improve community living and health outcomes for recipients of HCBS.

## Conclusion

The US may still be a long way from reckoning with the need for a comprehensive, well-funded system to provide LTSS. But as the population quickly ages and the pandemic creates millions more people who need LTSS and cannot rely on family members to fill in the gaps, there is an urgent need and opportunity to strengthen the piecemeal systems already in place. Medicaid HCBS continue to expand and evolve, and the need for a robust, multi-layered, beneficiary-centered oversight and accountability system has never been greater. Many new tools are just coming on line to vastly improve states' ability to evaluate HCBS quality, but states need resources and impetus from the federal government to make meaningful use of these measures to enhance the quality of HCBS for individuals with disabilities and older adults.

## Author Contributions

All authors listed have made a substantial, direct, and intellectual contribution to the work and approved it for publication.

## Funding

This study was supported by the National Institute on Disability, Independent Living, and Rehabilitation Research, grant number 90TRCP0004. NIDILRR is a Center within the Administration for Community Living (ACL), U.S. Department of Health and Human Services (HHS).

## Conflict of Interest

The authors declare that the research was conducted in the absence of any commercial or financial relationships that could be construed as a potential conflict of interest.

## Publisher's Note

All claims expressed in this article are solely those of the authors and do not necessarily represent those of their affiliated organizations, or those of the publisher, the editors and the reviewers. Any product that may be evaluated in this article, or claim that may be made by its manufacturer, is not guaranteed or endorsed by the publisher.
